# Recent Assembly of an Imprinted Domain from Non-Imprinted Components

**DOI:** 10.1371/journal.pgen.0020182

**Published:** 2006-10-27

**Authors:** Robert W Rapkins, Tim Hore, Megan Smithwick, Eleanor Ager, Andrew J Pask, Marilyn B Renfree, Matthias Kohn, Horst Hameister, Robert D Nicholls, Janine E Deakin, Jennifer A. Marshall Graves

**Affiliations:** 1 Australian Research Council Center for Kangaroo Genomics and Research School of Biological Sciences, Australian National University, Canberra, Australia; 2 Department of Genetics, La Trobe University, Melbourne, Australia; 3 Department of Zoology, University of Melbourne, Melbourne, Australia; 4 Department of Medical Genetics, University of Ulm, Ulm, Germany; 5 Department of Pediatrics, Children's Hospital of Pittsburgh, Pittsburgh, Pennsylvania, United States of America; The Babraham Institute, United Kingdom

## Abstract

Genomic imprinting, representing parent-specific expression of alleles at a locus, raises many questions about how—and especially why—epigenetic silencing of mammalian genes evolved. We present the first in-depth study of how a human imprinted domain evolved, analyzing a domain containing several imprinted genes that are involved in human disease. Using comparisons of orthologous genes in humans, marsupials, and the platypus, we discovered that the Prader-Willi/Angelman syndrome region on human Chromosome 15q was assembled only recently (105–180 million years ago). This imprinted domain arose after a region bearing *UBE3A* (Angelman syndrome) fused with an unlinked region bearing *SNRPN* (Prader-Willi syndrome), which had duplicated from the non-imprinted *SNRPB/B′.* This region independently acquired several retroposed gene copies and arrays of small nucleolar RNAs from different parts of the genome. In their original configurations, *SNRPN* and *UBE3A* are expressed from both alleles, implying that acquisition of imprinting occurred after their rearrangement and required the evolution of a control locus. Thus, the evolution of imprinting in viviparous mammals is ongoing.

## Synopsis

Humans and other mammals have two copies of the genome. For most genes, both copies are active. However, some genes are active only when they are inherited from the father, others only when inherited from the mother. These “imprinted” genes are clustered in domains that are controlled coordinately. Only mammals show genomic imprinting. It is not understood how or why genes became imprinted during mammalian evolution. The authors used comparisons between humans and the most distantly related mammals, marsupials and monotremes, to discover how one of these imprinted domains evolved. The authors studied an imprinted domain on human Chromosome 15, mutations which cause Prader-Willi and Angelman syndromes (PWS-AS). They discovered that the PWS and AS genes lie on different chromosomes in kangaroos and platypus and are not imprinted. Other imprinted genes in the domain, including the putative control region, are absent from the genome and derived from copies of genes from yet other chromosomes. The arrangement in kangaroos and platypus is present also in the chicken genome, so it must be ancestral. This study concludes that the PWS-AS imprinted region was assembled relatively recently from non-imprinted components that were moved together or copied from all over the genome.

## Introduction

Genomic imprinting refers to the silencing of a gene or region according to its parent of origin. Among vertebrates, imprinting is specific to mammals, with about 83 mammalian genes shown to be imprinted. About half are paternally expressed (that is, the maternally derived allele is suppressed) and half maternally expressed (the paternally derived allele is suppressed) [1]. Many genes subject to imprinting are involved with either developmental disorders or cancer (sometimes both), so understanding how and why genomic imprinting evolved and how it functions is therefore of compelling interest to medicine as well as biology.

Imprinting is an important model system for studying epigenetic regulation—an accelerating field of biology that focuses on how identical DNA sequences are differentially expressed to produce different phenotypes. The molecular mechanism of imprinting resembles X chromosome inactivation in females, another mammal-specific epigenetic phenomenon, suggesting that X inactivation and autosomal imprinting may share a common origin [2,3].

We do not yet understand what selective forces eschewed the benefits of diploidy in favor of parental imprinting, and there are many hypotheses to account for the seemingly perverse evolution of hemizygosity at these loci. Perhaps the most interesting and widely debated is the parental conflict hypothesis [4], now developed into the kinship hypothesis (reviewed [5]), which proposes that imprinting evolved in response to the antagonistic interests of parental genomes.

The origin, as well as the mechanism, of imprinting can be investigated by comparing gene arrangement and expression between divergent species. The observation that genes imprinted in human and mouse *(IGF2, M6P/IGF2R)* are not imprinted in chicken [6,7] implies that imprinting is specific to mammals. Nonetheless, the gene content and arrangement of human imprinted domains is highly conserved in chicken [8] and other vertebrates. For instance, the content and arrangement of coding genes in the human Beckwith-Wiedemann imprinted cluster (including *IGF2, H19, ASCL2, KCNQ1,* and *CDKN1C*) is largely shared with birds and fish, but the non-coding regulatory *H19* RNA is missing, along with large stretches of repetitive sequences and retroelements and several sequences thought to exert local control of imprinting [9,10].

The transition of a region from a non-imprinted state in fish and chicken to an imprinted state in placental mammals could therefore be dissected by comparing orthologous regions with the most divergent mammal groups. Marsupials and monotremes diverged from placental mammals 180 and 210 million years ago (MYA), respectively [11], so fill the 310-MY evolutionary void that separates birds and reptiles from humans and mice. This permits the reconstruction of gene content and arrangement over a long evolutionary period and provides informative sequence comparisons with high signal to noise ratios [12]. Importantly, these “alternative mammals” represent the transition between egg-laying and viviparous animals. Monotremes lay eggs, like reptiles. Marsupial young are born at an early developmental stage and complete development attached to a teat (often protected in a pouch). These modes of reproductive strategy represent major differences in the level of maternal investment, as well as the ability of paternally derived genes to influence maternal resources.

Genomic imprinting has been demonstrated in marsupials for *IGF2* [6], *PEG1*/*MEST* [13], and *IGF2R* [14]. However, *IGF2* and *IGF2R* show biallelic expression in monotremes [14,15]. A comparative study, specifically of the non-imprinted *IGF2* in platypus with the imprinted opossum, mouse, and human locus, reveals that the absence of *cis*-acting elements, such as short interspersed transposable elements and an intergenic conserved inverted repeat containing putative CTCF-binding sites, may be important for *IGF2* imprinting [16].

The occurrence of imprinting in marsupials, but not monotremes, would date the emergence of genomic imprinting in vertebrates to after 210 MYA, when therian mammals diverged from the egg-laying monotremes and before the divergence of marsupials and placentals 180 MYA. These limited data are consistent with the hypothesis that imprinting evolved after viviparity, as would be expected if it is selected as a response to parental conflict. However, this important conclusion is rather tenuous, since it is based upon expression data from only three of the ~80 genes imprinted in placental mammals [17].

We have therefore made a detailed comparison of the arrangement and expression of orthologs of another cluster of imprinted genes in the three major mammal groups (placentals, marsupials, and monotremes) and other vertebrates.

**Figure 1:**
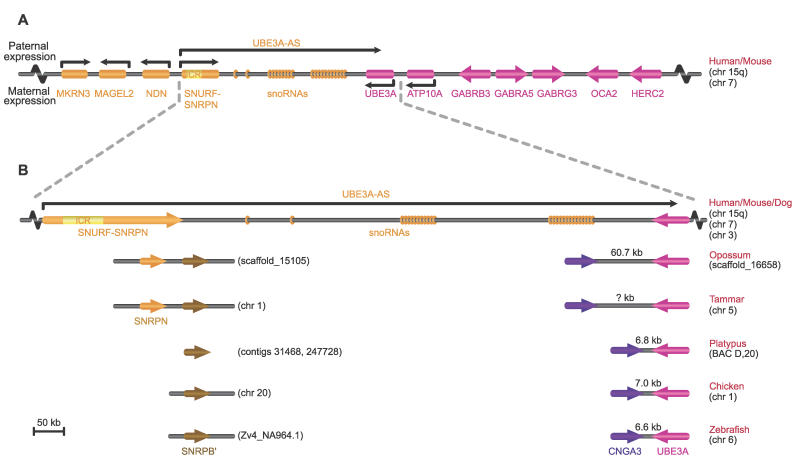
Genes of the PWS-AS Imprinted Domain (A) The PWS-AS imprinted domain in human and mouse. Paternal or maternal expression is indicated by arrows. The yellow region of *SNURF/SNRPN* represents the ICR. *UBE3A*-*AS* is an antisense transcript that includes arrays of untranslated snoRNA genes. (B) Comparison of the eutherian *SNRPN*–*UBE3A* region with its ancestral arrangement in non-eutherian vertebrates. Pink represents genes that are co-linear in humans through to fish, purple represents human Chromosome 2 genes that are adjacent to the PWS-AS homologous region in the ancestral arrangement, orange represents eutherian-specific genes or elements, and brown the unlinked gene *SNRPB′* that duplicated to form *SNRPN*. In marsupials, monotremes, chicken, and fish, *UBE3A* lies close to a human Chromosome 2 gene *CNGA3,* and there are no snoRNAs. In zebrafish, chicken, and platypus, only *SNRPB′* is present, but in marsupials tandem duplication gave rise to *SNRPN,* which was relocated next to *UBE3A* in eutherians.

Prader-Willi and Angelman syndromes (PWS and AS) are phenotypically distinct disorders associated with abnormalities (usually deletions) of a cluster of imprinted genes on human Chromosome 15q11-q13 [18] that amongst other things, influence feeding behavior. The regulation of imprinted genes in the PWS-AS domain has been studied in detail in humans and mice. The region comprises the AS and PWS domains (Figure 1A), within which deletions cause one or another disease. In the distal AS region lie two genes, *UBE3A* (thought to be solely responsible for AS) and *ATP10A,* both maternally expressed (paternally silenced) in brain [19–22]. The larger, more proximal PWS domain encompasses five paternally expressed (maternally silenced) genes responsible for Prader-Willi syndrome, including *SNRPN,* which encodes the SmN antigen. A large paternal transcript originating upstream of the *SNURF-SNRPN* genes liberates several classes of small nucleolar RNAs (snoRNAs) [23]. The imprinted expression of the AS and PWS domains is orchestrated by a bipartite imprinting control region (ICR) located within a 35-kilobase (kb) region which encompasses the *SNRPN* promoter [24,25]. Splice variants of the *SNURF-SNRPN* transcript, which are anti-sense to *UBE3A,* may provide the regulatory link between the ICR (in the PWS domain) and the imprinted genes of the AS domain [26].

The evolutionary history of the PWS-AS imprinted region is unknown, although recent retrotranspositions into the mouse domain have been noted [27]. We therefore cloned and characterized marsupial and monotreme orthologs of genes in the human 15q11-q13 region. To our astonishment, we discovered that marsupial and monotreme AS and PWS genes lie on different chromosomes, and we used bioinformatic analysis to show that this constitutes the ancestral arrangement. Both genes are biallelically expressed in marsupials and monotremes. Other genes from the PWS-AS region are absent from the marsupial and monotreme genomes. Thus, rearrangement of PWS-AS genes and acquisition of retrotransposed genes and key regulatory elements occurred much later in placental mammals, allowing their coordinate imprinted expression.

## Results

We examined the evolutionary origin of genes in and adjacent to the human PWS-AS imprinted region by isolating, mapping, and assessing transcription of their orthologs in marsupials, platypus, chicken, and fish.

### Isolation of Marsupial and Monotreme Homologs of PWS-AS Genes

We screened bacterial artificial chromosome (BAC) and cDNA libraries from the model kangaroo Macropus eugenii (tammar wallaby) for several human 15q11-q13 genes. cDNA clones containing *GABRB3* and *HERC2* and two BACs containing the AS gene *UBE3A* were confirmed by partial sequencing.

Numerous attempts over several years to isolate other imprinted genes, using PCR amplification, Southern blot analysis, and screening several genomic and cDNA libraries, were consistently unsuccessful. Screening for *MKRN3* identified a hitherto unknown intron-containing source gene makorin *(MKRN1)* [28]. Our conclusion that the marsupial genome lacks *MKRN3* was supported by its absence from the Monodelphis domestica (opossum) database. No clones containing tammar *MAGEL2* or *NDN* were ever recovered, and no *MAGEL2* or *NDN* sequences were found in the opossum database.

Similarly, attempts to clone tammar homologs of the imprinted PWS gene *SNRPN* from a genomic DNA lambda library resulted in the repeated isolation of a non-imprinted paralog *SNRPB/B′* [29], which lies on human Chromosome 20. Subsequently, we discovered a second *SNRPN-*like transcript encoding a predicted gene product that clusters with the SmN proteins, demonstrating orthology to *SNRPN* (Figure S1). A single tammar BAC was then obtained containing both *SNRPN* and *SNRPB′* homologs. Searching the opossum draft assembly for this sequence located a *SNRPN* ortholog directly adjacent to *SNRPB′* on scaffold 15105. Neither this scaffold, nor any other opossum sequence contained sequences with homology to human *SNURF.* We therefore conclude that the marsupial genome contains tandemly arranged *SNRPN-SNRPB′* sequences, and that *SNURF* is absent.

To explore still more ancient arrangements of these genes, we screened a platypus BAC library for PW-AS orthologs. No *SNRPN* homolog was ever obtained. Sequence retrieved from the platypus trace archive confirmed the presence of *SNRPB′,* but not *SNRPN.* A platypus BAC containing *UBE3A* was obtained and its identity confirmed by sequencing. Surprisingly, full sequencing of this BAC also identified the ortholog of a human Chromosome 2 gene, *CNGA3,* 6.8 kb from *UBE3A* in a tail-to-tail arrangement.

To determine whether *UBE3A* is adjacent to *CNGA3* also in marsupials, we screened the two tammar *UBE3A* BACs for *CNGA3.* We identified *CNGA3* in the larger BAC and confirmed its presence by sequencing. We also searched the opossum draft assembly, finding that *UBE3A* and *CNGA3* lie ~60 kb apart in a tail-to-tail arrangement on scaffold 16658, which also contained flanking genes from human Chromosomes 15q and 2 (Table 1).

**Table 1:**
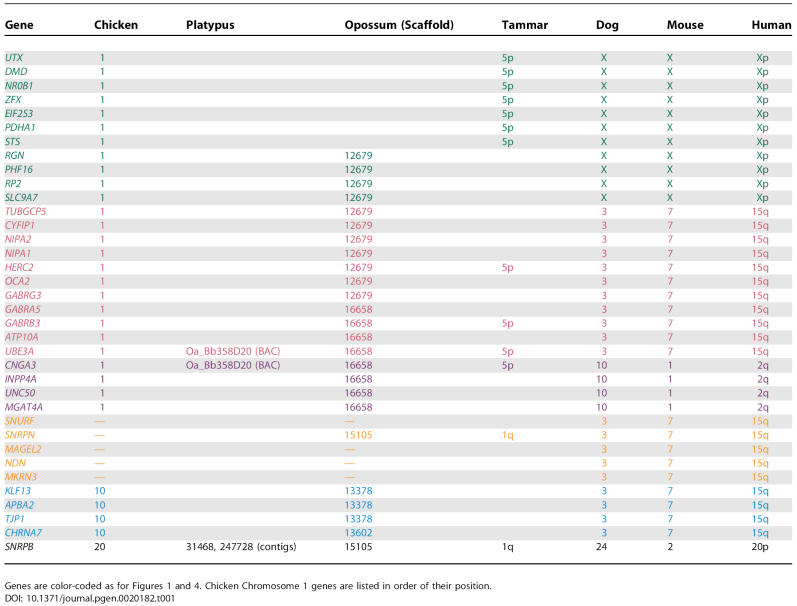
Positions of Orthologs of Human PWS-AS Genes and Their Flanking Markers in Chickens and Three Groups of Mammals

### Mapping PWS-AS Genes in Marsupials

**Figure 2:**
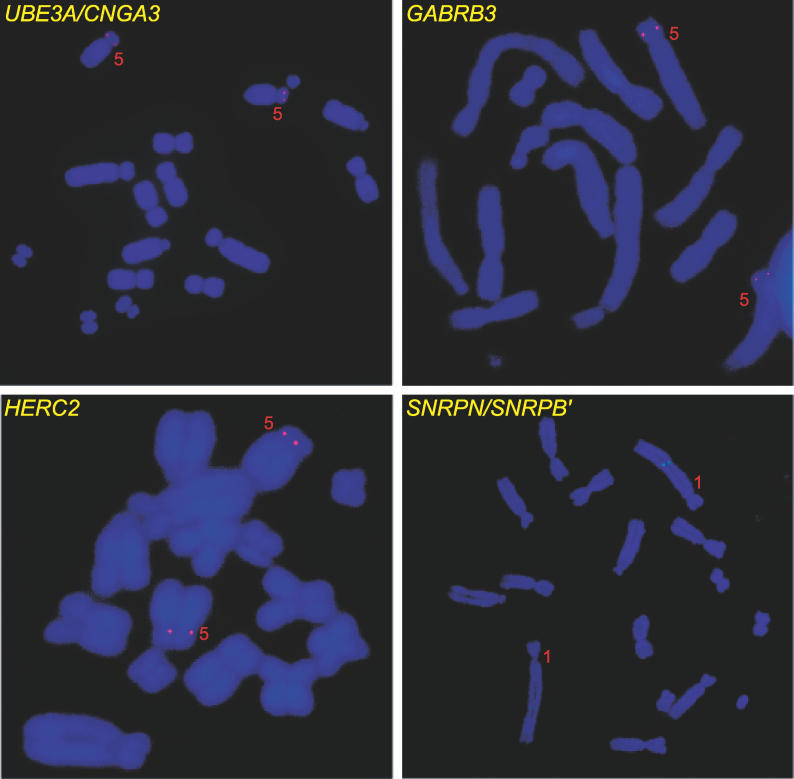
FISH Localization of BACs Containing Tammar Homologs of PWS-AS Genes *UBE3A*/*CNGA3, GABRB3,* and *HERC2* localize to tammar wallaby Chromosome 5p and *SNRPN*/*SNRPB* to Chromosome 1q.

We mapped tammar PWS-AS genes by fluorescence in situ hybridization (FISH) to determine whether their co-location on human 15q is conserved in marsupials. *GABRB3* and *HERC2* (lambda clones), as well as the BAC containing *UBE3A* and *CNGA3* co-localized in the middle of tammar Chromosome 5p (Figure 2). This was unexpected because tammar 5p has homology to the short arm of the human X chromosome (reviewed [30]). This location was also different to the localization on tammar 1q of a lambda clone originally thought to contain *SNRPN* [31], but now identified as *SNRPB′.* We mapped the tammar BAC containing both *SNRPN* and *SNRPB′* unequivocally to the middle of tammar Chromosome 1q (Figure 2).

Thus, the arrangement of marsupial and monotreme orthologs of PWS-AS genes differs from that in humans and mice. *UBE3A* and flanking genes do not co-localize with *SNRPN,* but instead share synteny with genes on human Chromosomes Xp and 2. To determine which arrangement is ancestral, we extended our comparison to other vertebrates, making use of information in public databases.

### Other Vertebrate Genomes Reveal the Ancestral Arrangement of PWS**-**AS Genes

We searched the UCSC database (http://www.genome.ucsc.edu) for dog, chicken, and fish homologs of genes in and near the human PWS-AS region, including *UBE3A, GABRB3,* and *HERC2* (tammar PWS-AS orthologs), as well as *CNGA3* and genes adjacent to this region in tammar (lying on Xp in human). Orthologs of these genes all lay on scaffolds mapping to chicken Chromosome 1 (Table 1). A block of nine human 15q11-q13 genes (from *UBE3A* to the centromeric *TUBGCP5*) was found sandwiched between large blocks of human Xp and Chromosome 2 genes on chicken Chromosome 1. *CNGA3,* flanking a block of human Chromosome 2 genes, lies only 2 kb from *UBE3A.* In zebrafish, although the synteny groups are duplicated and somewhat broken up, *UBE3A* and *CNGA3* lie on Chromosome 6 along with several other genes from the human X, 15, and 2 blocks and are separate from genes that flank this region in human, which lie on Chromosomes 7 and 20.

Attempts to retrieve *SNURF*-*SNRPN* and *MKRN3* sequence from the chicken and zebrafish databases yielded only the ancestral *SNRPB′* and *MKRN1,* suggesting that non-mammal vertebrates lack *SNURF*-*SNRPN* and *MKRN3.* No sequences orthologous to *MAGEL2* and *NDN* were found in chicken and fish genomes (Table S2).

The finding that human 15q11-q13 genes lie between blocks of human Xp and Chromosome 2 genes in chicken and fish, as well as marsupials and monotremes, and that *MKRN3, MAGEL2, NDN,* and *SNURF* are absent, implies that this is an ancestral vertebrate arrangement. Its occurrence also in marsupials and monotremes implies that the ancestral arrangement was retained in marsupials and monotremes, but the region was rearranged and augmented more recently in placental mammals.

### Are *SNRPN* and *UBE3A* Imprinted in Marsupials and Monotremes?

Of the greatest interest was to determine whether marsupial and monotreme *SNRPN* and *UBE3A* are imprinted like their human orthologs, even though they do not share the gene arrangement of the placental PWS-AS region.

**Figure 3:**
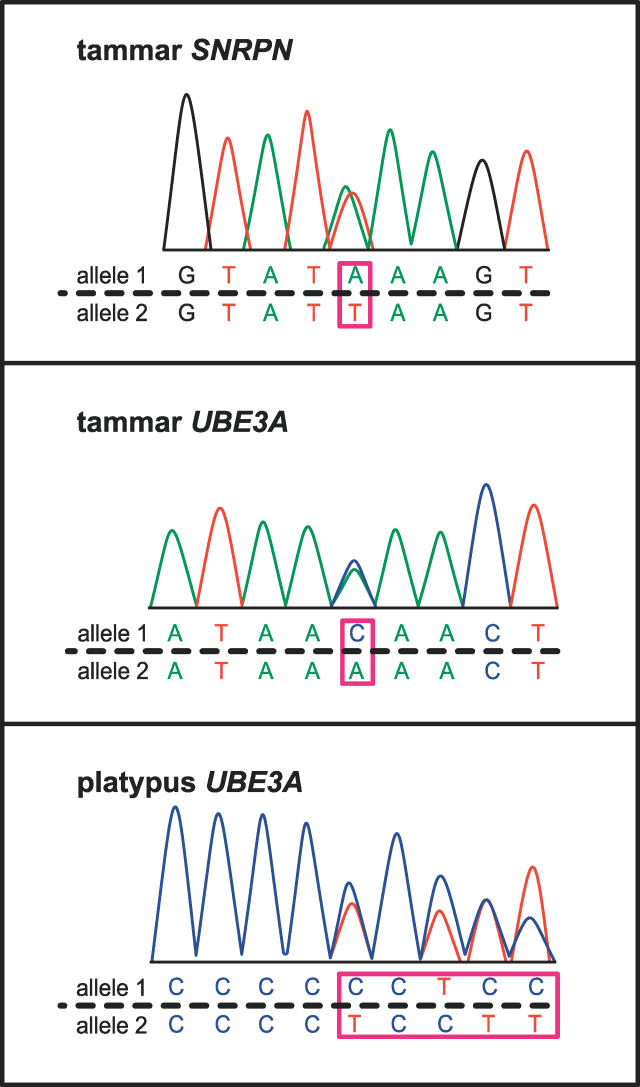
Biallelic Expression Demonstrated by Sequencing Brain cDNA from Heterozygous Animals for Alleles of *SNRPN* and *UBE3A* in Tammar Wallaby and *UBE3A* in Platypus Alleles differ at an A/T polymorphism at base pair 67 of the 3′ UTR of tammar *SNRPN,* a C/T polymorphism at base pair 247 of exon 5 in tammar *UBE3A,* and an insertion polymorphism of a C at base pair 179 of the 3′ UTR in platypus *UBE3A* (marked by boxes).

Expressed polymorphisms in these two genes in marsupials were therefore sought by screening DNA from 60 tammar wallabies. Sequence analysis revealed a *SNRPN* polymorphism in one heterozygote and a *UBE3A* polymorphism in two. Brain RNA (the tissue showing imprinting of both *UBE3A* and *SNRPN* in placental mammals) was extracted from these animals and a fragment amplified from the resulting cDNA template using gene-specific primers that spanned introns. Sequencing the PCR products revealed expression at similar levels of both parental alleles for both genes (Figure 3).

We also investigated the expression of monotreme *UBE3A* (but not *SNRPN,* since it appears to be absent from the monotreme genome). DNA from five platypuses was screened, and a *UBE3A* polymorphism detected in one heterozygous animal, from which brain tissue was isolated and RNA extracted. Amplification and sequencing from brain cDNA revealed expression of both alleles (Figure 3). Because the alleles differed by a base pair insertion, this result was confirmed by primer extension assays (Figure S2).

Expression studies therefore show that *UBE3A* (the Angelman syndrome gene) is biallelically expressed in marsupial and monotreme brain. The Prader-Willi gene *SNRPN,* represented by a tandem duplication of the non-imprinted gene *SNRPB′* in marsupials, also does not appear to be imprinted.

## Discussion

Our results demonstrate that the PWS-AS imprinted region of placental mammals was assembled from a variety of disparate genomic elements and suggest that imprinting was acquired relatively recently in the placental lineage. These conclusions follow from differences in arrangement and expression of *SNRPN* and *UBE3A* in the three major mammal groups, placentals, marsupials, and monotremes.

*SNRPN* and *UBE3A* lie together in all placental mammals investigated, including the fully sequenced human, mouse, and dog. In the basal placental clades, Afrotheria and Xenarthra, sequence assembly is not sufficiently advanced to ascertain gene arrangement, but chromosome painting experiments reveal that human Chromosome 15q is represented as a single block in elephant and armadillo [32,33]. In contrast, we demonstrate that the two primary PWS-AS loci *SNRPN* and *UBE3A* lie on different chromosomes in marsupials and monotremes, and that other PWS-AS loci have no orthologs in marsupials or monotremes. Since this gene arrangement is shared by birds and fish, it must be ancestral.

**Figure 4:**
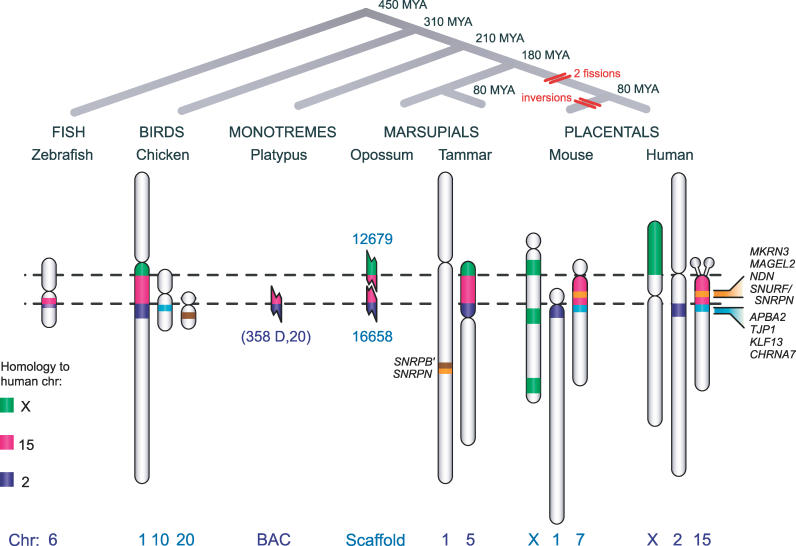
Assembly of the PWS-AS Imprinted Region in Placental Mammals during Vertebrate Evolution Relationships between fish, birds/reptiles, and the three mammal groups are presented as a phylogeny (top). In the ancestral arrangement, shared by marsupials, monotremes, birds, and fish, the block of imprinted human 15q genes (pink) is flanked by human X (green) and human 2 (purple) blocks. These three blocks were separated by at least two fissions and were rearranged next to an unlinked block of genes (on chick Chromosome 10, pale blue) to make up the present regions of human Chromosome 15q (and with two more inversions, of mouse Chromosome 7). *SNRPB′* (brown) is present on a different chromosome in fish, birds, and marsupials, but its duplicate *SNRPN* (orange) is transposed next to *UBE3A* in placentals. Other human 15q genes absent from non-placental vertebrates (orange) were independently added to the imprinted region in the placental lineage.

This implies that a major rearrangement occurred in the placental lineage to unite *UBE3A* and *SNRPN.* The evolutionary breakpoints lie between *SLC9A7* and *TUBGCP5* and between *UBE3A* and *CNGA3* in the ancestral sequence. Fission between *UBE3A* and *CNGA3* was evidently preceded by expansion of the interval from ~7.0 kb (chicken, fish, and monotremes) to >60 kb (marsupials) (Figure 1B). The *UBE3A* region then fused with an ancestral region represented by chicken Chromosome 10 that bears homologs of genes in human 15q11-q13 and flanking genes located distal to *HERC2* (Figure 4).

The presence of *MKRN3, MAGEL2,* and *NDN* in the genomes of dogs, mice, and humans, but their absence from chicken, fish, and the recently sequenced opossum and platypus genomes (each of which are 6× coverage or greater), implies that these genes were all acquired by the placental PWS-AS domain 180–90 MYA. They arrived independently by retrotransposition from paralogs on different chromosomes. The intronless *MKRN3* arose by retrotransposition from the intron-containing source gene *MKRN1* on human Chromosome 20 [28], and the intronless *MAGEL2* and *NDN* arose by retrotransposition from genes on different sites on the X. Since no snoRNA arrays lie near the ancestral *UBE3A* or *SNRPN,* these must also have been seeded from other locations. Thus, the expanded and fused region was evidently a target for insertions and rearrangements. It seems to have remained unstable, since the mouse and human regions differ by several retrotranspositions [27], and the region contains breakpoint hotspots that are the source of many PWS and AS deletions [34].

We conclude from our expression studies that *UBE3A* and *SNRPN* are biallelically expressed in marsupial and monotreme brain. Because imprinted expression of *UBE3A* is found within neurons, but not glial cells of the brain [35], it is possible that we could not detect some level of allelic attenuation for *UBE3A* in marsupials and monotremes. However, considering that the brain of AS patients produces only about 10% *UBE3A* expression [22], we consider that even semi-quantitative methods such as direct sequencing should have detected this level of imprinting in marsupial and monotreme brain. Our finding that *SNRPN* and *UBE3A* are not imprinted in their original locations is consistent with the hypothesis that rearrangement was required for the establishment of imprinting.

Since *UBE3A* and *SNRPN* appear not to be imprinted in marsupials and monotremes, we would not expect to find sequences that control imprinting of these genes, so the absence of *SNURF* and the ICR from the marsupial genome is particularly telling. *SNURF* lies in the ICR (as defined by the region of shortest deletion in human PWS patients) and the *UBE3A* anti-sense transcript originates either at its 5′ end or at alternative upstream exons [36]. We propose that coordinate regulation of the PWS-AS domain required introduction of these sequences into the unstable fused region.

The time at which chromosome rearrangement occurred and imprinting was acquired can be deduced from the mammalian phylogeny (Figure 4). Gene arrangement in the PWS-AS domain is conserved at least between dog, mouse, and human, and the region was syntenic even in the most distantly related placentals, implying that it predated the placental radiation ~105 MYA. The ancestral arrangement in chicken and fish is shared by marsupials and monotremes, which diverged from placentals 180 and 210 MYA, respectively. Major rearrangements therefore occurred after placentals and marsupials diverged 180 MYA, but before the placental radiation 105 MYA. Our demonstration that *SNRPN* and *UBE3A* are also biallelically expressed in marsupials shows that imprinting evolved considerably later (180–80 MYA) in this region than, for instance, in the *IGF2* region. This is consistent with the view that imprinting evolved after the evolution of viviparity in therian mammals, but shows, also, that at least some loci evolved imprinting considerably after this time. The recent findings that the imprinted gene *Nnat* is eutherian-specific [37], and *DLK1* is not imprinted in the opossum [38], also suggest that novel genes could be imprinted more recently. Thus, imprinting has been acquired at different times in different domains, after the evolution of viviparity, and suggests that viviparity is a necessary but not a sufficient condition for the evolution of imprinting.

Is there any significance in our finding that the PWS-AS imprinted domain once shared synteny with the genome region that became part of the placental X and was recruited into the X inactivation system in placental mammals? There has been continuing speculation that genomic imprinting and X chromosome inactivation are related by descent [2,3]. One possibility is that the entire region was added to the ancestral therian X and recruited to the X inactivation system, and then the PWS-AS region was subsequently relocated to an autosome, carrying elements that control *cis*-silencing. This could also explain the acquisition by this human Chromosome 15 region of *MAGE* (cancer-testis antigen) genes, nearly all of which accumulated on the X chromosome in placentals [39].

In conclusion, we show that the Prader-Willi/Angelman imprinted domain on human Chromosome 15q11–13 was assembled relatively recently from unlinked and non-imprinted components in a mammalian ancestor. Two non-imprinted regions fused 105–180 MYA, and several retroposed genes and snoRNAs from different regions were independently inserted. We propose that genomic rearrangement early in the eutherian lineage was required for the acquisition of imprinting at this locus.

## Materials and Methods

### Tissue samples.

Tammar wallaby tissue samples were sourced at the Research School of Biological Sciences, Australian National University, Canberra, and the Department of Zoology, University of Melbourne, according to the Animal Experimentation Ethics Committee. Platypus samples were supplied courtesy of Dr. F. Grützner, Australian National University, Canberra, Australia.

### Nucleic acid extraction, amplification, and sequencing.

Total genomic DNA was extracted from tail clippings of 20 pouch young (py) tammar wallabies (collected June 2005), from the brain of three py tammars (collected November 2003), and from ear punches of 30 adult tammars (collected March 2005) using the DNeasy Tissue Kit (Qiagen, Valencia, California, United States) or the protocol outlined in [40]. Total genomic DNA was extracted from two male and one female platypus brain samples (collected September 2004). High-copy number plasmids were extracted using either the Wizard Plus SV Minipreps DNA Purification System (Promega, Madison, Wisconsin, United States) according to the manufacturer's instructions or by the protocol outlined by Sambrook [40]. BAC DNA was extracted using the Wizard Plus SV Minipreps DNA Purification System (Promega).

Total RNA was extracted from the brains of polymorphic platypus and tammar wallabies using the RNeasy Mini Kit (Qiagen) according to the manufacturer's instructions or by RNaWIZ RNA Isolation reagent. Total RNA was reverse-transcribed using the SuperScript III First-Strand Synthesis System for RT-PCR (Invitrogen, Carlsbad, California, United States) according to manufacturer's instructions.

PCR amplification was conducted on a MJ Research PTC-200 thermal cycler using a 20-μl reaction, including up to 200 ng of template DNA, 1 × PCR reaction buffer (Roche Applied Sciences, Basel, Switzerland), 200 μM dNTPs (Roche Applied Sciences), 0.8 μM of each forward and reverse primer (Table 3), and 1 unit of *Taq* (Roche Applied Sciences). The cycling conditions were 94 °C, 2 min; 35 × (94 °C, 30 s; 54–60 °C, 1 min; 72 °C, 2 min); 72 °C, 10 min.

DNA sequencing was carried out by the Washington University Genome Sequencing Center, St. Louis Missouri, United States or the Australian Genome Research Facility, Brisbane, Australia.

### DNA library screening.

50 ng of purified DNA probe was denatured in boiling water for 3 min and radioactively labeled with 50 μCi of [α-^32^P] dCTP using the Megaprime labeling kit (Amersham, Little Chalfont, United Kingdom) according to the manufacturer's instructions. To remove unincorporated nucleotides the labeled probe was run through a ProbeQuant G-50 Micro column (Amersham Pharmacia Biotech) according to the manufacturer's instructions or through a Sephadex G-50 (Pharmacia) column by centrifugation at 1,000 *g* for 1 min. Library screening using radioactive overgos was also undertaken on the platypus library as described previously [41]. Probes were hybridized to various DNA libraries (Table 2) and exposed to autoradiography film at −70 °C until sufficiently exposed.

**Table 2:**
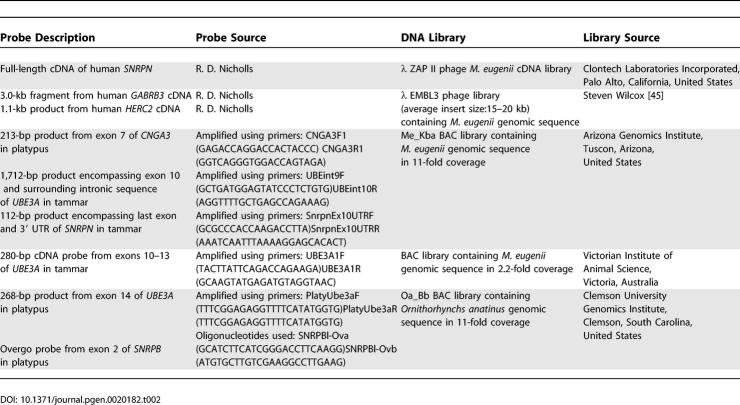
Description of Probes Used and DNA Libraries Screened

**Table 3:**
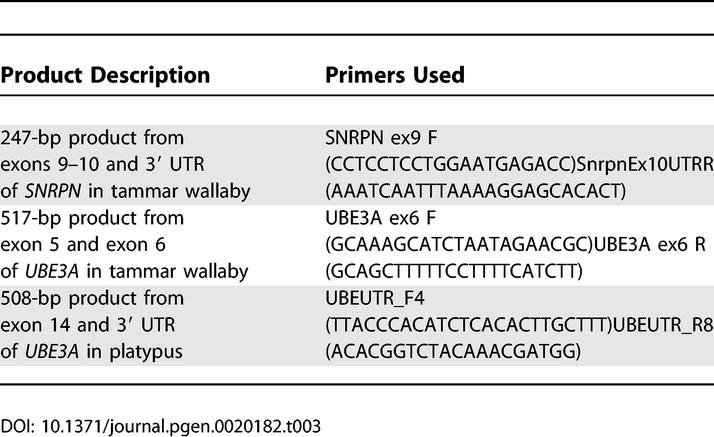
PCR Products Used in Expression Studies

### FISH.

Mitotic metaphase chromosomes were prepared from tammar wallaby fibroblast cell lines grown from ear punctures of adult male wallabies. FISH was carried out as previously described [31]. Probes were fluorescence-labeled by the BioNick Labeling System (Invitrogen) in accordance with the manufacturer's instructions. Probes were detected using fluorescein isothiocyanate (FITC)-conjugated avidin and biotin-conjugated anti-avidin antibody (Vector Laboratories, Burlingame, California, United States). Chromosomes and cell nuclei were counterstained with 1 μg/ml 4,6-diamidino-2-phenylindole (DAPI) in 2 X SSC for 1 min and mounted.

Fluorescence signals were visualized and captured using a Zeiss (Oberkochen, Germany) Axioplan epifluorescence microscope equipped with a CCD (charge-coupled device) camera (RT-Spot, Diagnostic Instruments, Sterling Heights, Michigan, United States). Images were manipulated using IPlab imaging software on an Apple Macintosh computer (Cupertino, California, United States). Gray scale images were captured with source images superimposed into color images.

### Expression analysis.

Tammar py heterozygous for an expressed polymorphism ranged in age from 104 d to 116 d. PCR products used in expression studies were amplified using primers shown in Table 3. PCR products were purified prior to sequencing using the QIAquick gel extraction kit (Qiagen). Single nucleotide primer extension (SNUPE) assays were undertaken on adult platypus samples using the MassARRAY analyzer system (Sequenom, San Diego, California, United States) by the staff at the Australian Genome Research Facility.

### Bioinformatic analysis.

Human protein sequence from genes of interest (Table S1) were extracted and interrogated against the mouse (assembly: March 2005), dog (assembly: May 2005), opossum (assembly: October 2004), chicken (assembly: February 2004), and zebrafish (June 2004) databases deposited on the UCSC genome browser (http://www.genome.ucsc.edu) using the BLAT algorithm with default settings [42] (Table S2). In an attempt to identify highly diverged homologs of proteins *SNURF, SNRPN, MAGEL2,* and *NDN* the more sensitive tBLASTn algorithm [43] was used on databases accessible through the Ensembl website (http://www.ensembl.org). No significant alignments in addition to those found using BLAT were produced. Sequence from the draft platypus genome assembly (version 5.0), related to *SNRPB,* was extracted from the Washington University Genome Sequencing Center (http://genome.wustl.edu/tools/blast) using tBLASTn.

Members of the Sm family of proteins (Table S3) were aligned using the ClustalW program (http://www.ebi.ac.uk/clustalw) with default parameters. Phylogenetic analysis was performed with the maximum parsimony method using PAUP* version 4.0 b 10 [44]. Gaps were treated as missing data. Most-parsimonious trees were searched using a heuristic strategy, starting trees were obtained via stepwise addition for tree-bisection-reconnection (TBR) branch swapping, one tree was held at each step during stepwise addition, and a maximum of 1,000 best trees were saved in each replicate. 1,000 replications were performed for the bootstrap analysis. A pictorial consensus tree was created using the Phylodendron tree printer (http://iubio.bio.indiana.edu/treeapp/treeprint-form.html).

## Supporting Information

Figure S1 Relationships between Vertebrate Sm Proteins Encoded by the Genes *SNRPN* (SmN) and *SNRPB/B′* (SmB) Generated Using Maximum Parsimony Analysis Marsupial (opossum and tammar) SmN sequences are sister to placental SmN and distant from vertebrate SmB. Support for tree topology is indicated by bootstrap values (1,000 replicates).
(200 KB PDF)

Figure S2 Primer Extension Assay Relative allelic concentrations of PCR products amplified from the *UBE3A* gene of platypus brain genomic DNA and cDNA for the three individuals sampled. Standard deviation indicated by error bars.
(200 KB PDF)

Table S1 Human Protein Sequences Used in Study (37 KB DOC)

Table S2 Location of Putative Orthologs Aligned to Human Proteins of Interest in Mouse, Dog, Opossum, Tammar Wallaby, Platypus, Chicken, and Zebrafish Percent identity between query and target sequence is given, along with the coverage of this alignment relative to query sequence length. An asterisk (*) denotes that no significant alignment was found. % Id, percent identity; % cov, percent coverage.
(207 KB DOC)

Table S3 Sm Protein Family Members Used in Phylogenetic Studies and Their Accession Numbers (21 KB DOC)

Text S1 Expression Studies A primer extension assay was used to confirm the biallelic expression of *UBE3A* in platypus brain (Figure S2), because the polymorphism identified was a deletion and therefore difficult to test using direct sequencing. DNA was amplified in triplicate from genomic and cDNA samples extracted from the brain of three platypus individuals. One individual was homozygous for the C allele (I), another homozygous for the deleted allele (II), and a third heterozygous for both alleles (III). Each sample was subjected to a primer extension assay capable of quantifying the relative amount of each allele. In accordance with sequencing data, the heterozygous individual showed biallelic expression of the polymorphic site (Figure 1). Interestingly, there appeared to be an unexpected increase in the concentration of the deleted allele for all samples. This is most likely to be an experimental artifact, due to the polymorphism being located within a poly-C tract and resulting in primer slippage.
(20 KB DOC)

## Abbreviations

BAC: bacterial artificial chromosome
FISH: fluorescence in situ hybridization
ICR: imprint control region
kb: kilobase
MYA: million years ago
PWS-AS: Prader-Willi/Angelman syndrome
snoRNA: small nucleolar RNA
