# Supplementary figures and images for: Recent Assembly of an Imprinted Domain from Non-Imprinted Components
Source: PLoS Genet. 2006 Oct 27;2(10):e182. doi: 10.1371/journal.pgen.0020182 (PMC1626109; doi:10.1371/journal.pgen.0020182)

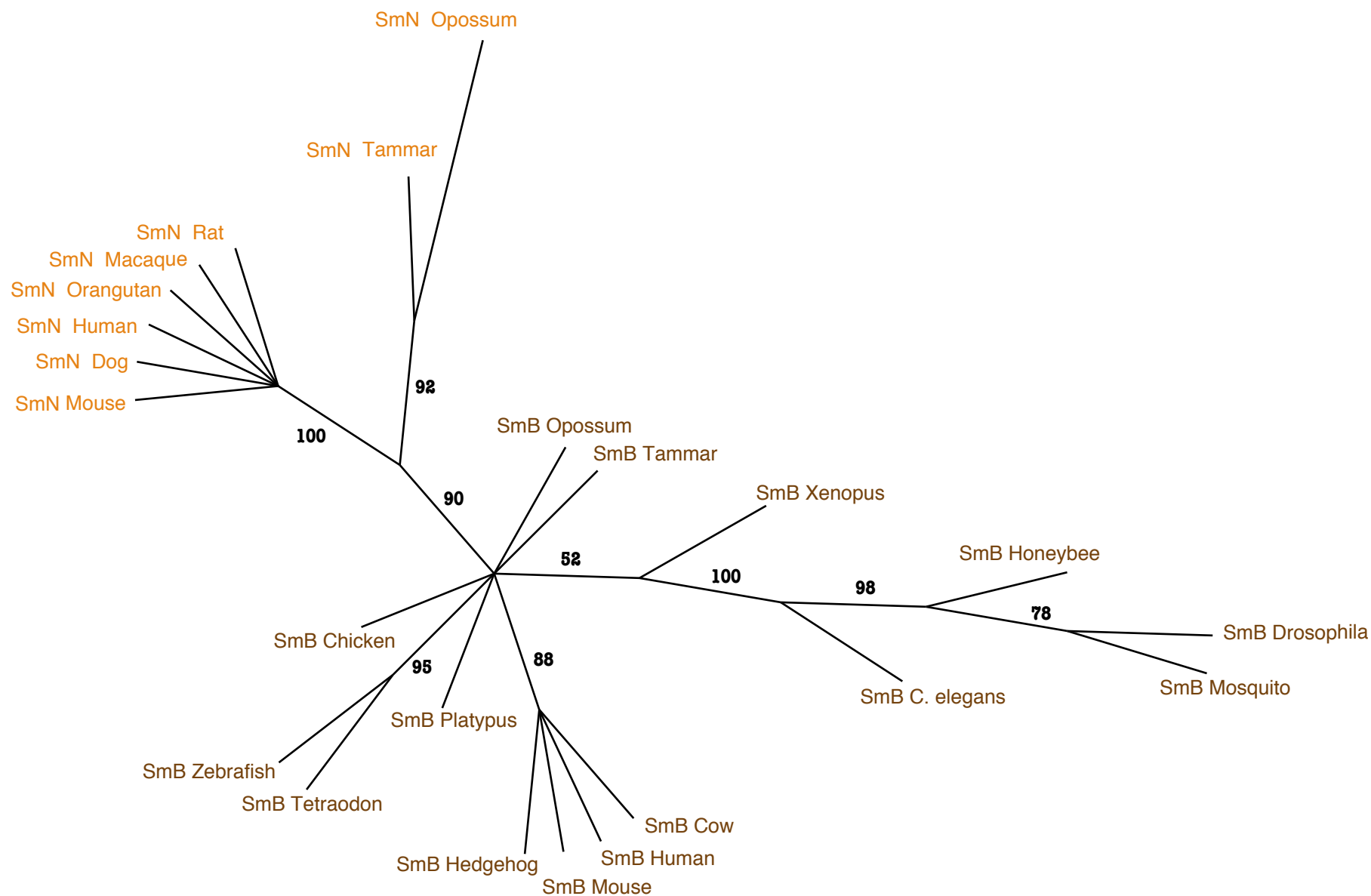

Supplement: Figure S1 — Marsupial (opossum and tammar) SmN sequences are sister to placental SmN and distant from vertebrate SmB. Support for tree topology is indicated by bootstrap values (1,000 replicates). (200 KB PDF) [file pgen.0020182.sg001.pdf]

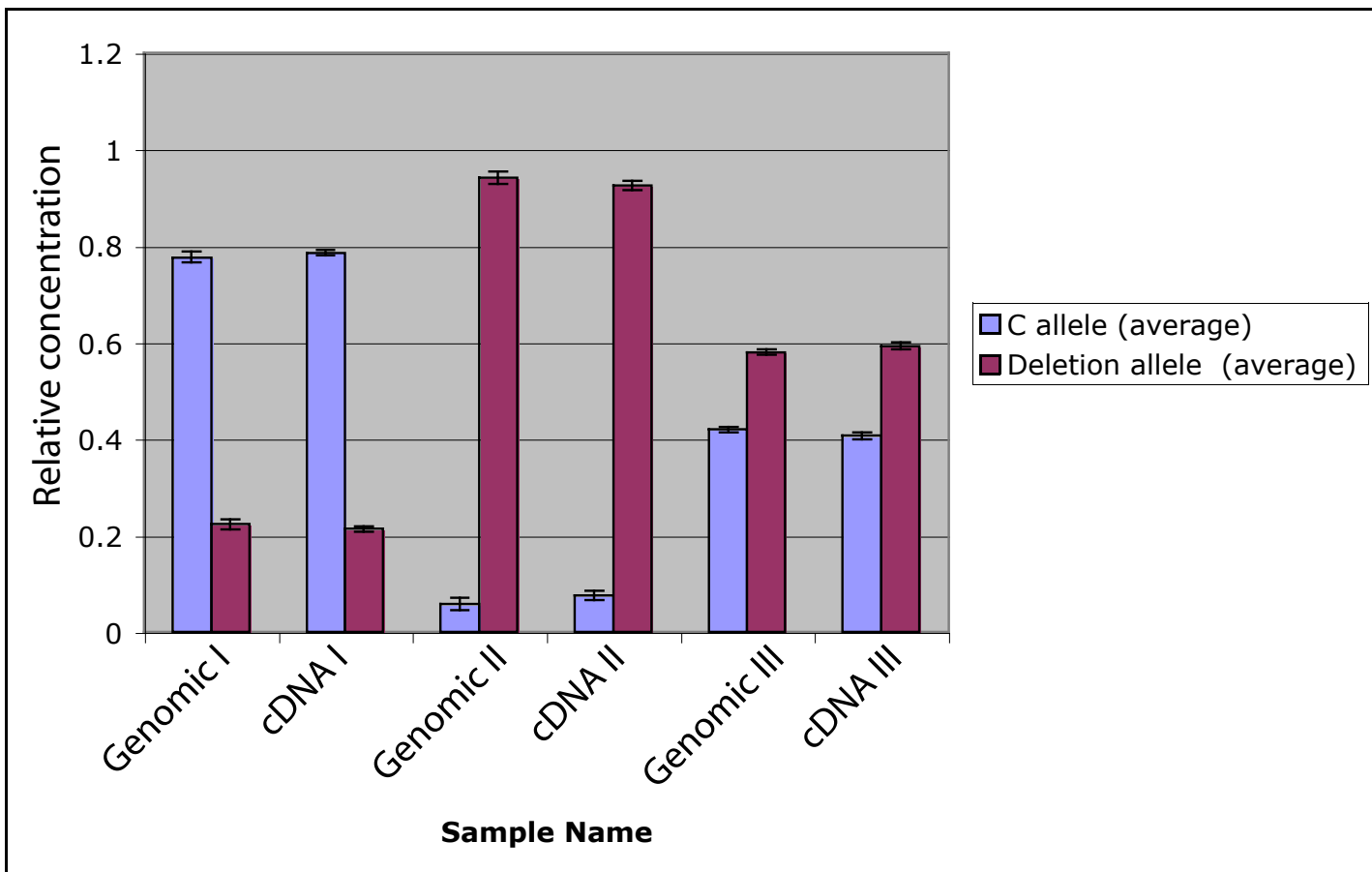

Supplement: Figure S2 — Relative allelic concentrations of PCR products amplified from the UBE3A gene of platypus brain genomic DNA and cDNA for the three individuals sampled. Standard deviation indicated by error bars. (200 KB PDF) [file pgen.0020182.sg002.pdf]
