# Supplementary material for: Recent Assembly of an Imprinted Domain from Non-Imprinted Components
Source: PLoS Genet. 2006 Oct 27;2(10):e182. doi: 10.1371/journal.pgen.0020182 (PMC1626109; doi:10.1371/journal.pgen.0020182)
Supplement: Table S1 — (37 KB DOC) [file pgen.0020182.st001.doc]

| Human |  |  |  |  |
| --- | --- | --- | --- | --- |
| Query | Accession number | Chromosome | Start | End |
| UTX | O15550 | chrX | 44489051 | 44725748 |
| DMD | P11532 | chrX | 30911873 | 32797972 |
| NR0B1 | P51843 | chrX | 30082355 | 30087137 |
| ZFX | P17010 | chrX | 23950516 | 23989147 |
| EIF2S3 | P41091 | chrX | 23832742 | 23854556 |
| PDHA1 | P08559 | chrX | 19121806 | 19137425 |
| STS | P08842 | chrX | 7030970 | 7128035 |
| RGN | Q15493 | chrX | 46696797 | 46708597 |
| PHF16 | Q92613 | chrX | 46600549 | 46674730 |
| RP2 | O75695 | chrX | 46452789 | 46495455 |
| SLC9A7 | Q96T83 | chrX | 46222643 | 46374721 |
| TUBGCP5 | Q96RT8 | chr15 | 20384959 | 20424686 |
| CYFIP1 | NP_055423 | chr15 | 20477223 | 20554478 |
| NIPA2 | NP_001008892 | chr15 | 20557664 | 20572777 |
| NIPA1 | NP_653200 | chr15 | 20600272 | 20637852 |
| UBE3A | Q05086 | chr15 | 23135379 | 23172002 |
| ATP10A | NP_077816.1 | chr15 | 23475583 | 23659336 |
| GABRB3 | P28472 | chr15 | 24344035 | 24569202 |
| GABRA5 | P31644 | chr15 | 24665488 | 24776123 |
| GABRG3 | Q99928 | chr15 | 24799428 | 25451619 |
| OCA2 | Q04671 | chr15 | 25674131 | 26000615 |
| HERC2 | NP_004658 | chr15 | 26030506 | 26240174 |
| APBA2 | Q99767 | chr15 | 27133379 | 27196625 |
| TJP1 | Q07157 | chr15 | 27781093 | 27880188 |
| KLF13 | Q9Y2Y9 | chr15 | 29406707 | 29451791 |
| CHRNA7 | P36544 | chr15 | 30110089 | 30247948 |
| CNGA3 | Q16281 | chr2 | 98444956 | 98472233 |
| INPP4A | NP_001557 | chr2 | 98595029 | 98662586 |
| UNC50 | NP_054763 | chr2 | 98684740 | 98693282 |
| MGAT4A | NP_036346 | chr2 | 98700703 | 98801319 |
| SNRPB | P14678 | chr20 | 2391279 | 2396404 |
| MKRN3 | Q13064 | chr15 | 21362022 | 21363543 |
| MAGEL2 | NP_061939.1 | chr15 | 21440235 | 21441795 |
| NDN | Q99608 | chr15 | 21482494 | 21483457 |
| SNURF | NP_073715 | chr15 | 22758351 | 22764274 |
| SNRPN | NP_003088.1 | chr15 | 22771597 | 22774681 |
