# Supplementary material for: Recent Assembly of an Imprinted Domain from Non-Imprinted Components
Source: PLoS Genet. 2006 Oct 27;2(10):e182. doi: 10.1371/journal.pgen.0020182 (PMC1626109; doi:10.1371/journal.pgen.0020182)
Supplement: Table S2 — Percent identity between query and target sequence is given, along with the coverage of this alignment relative to query sequence length. An asterisk (*) denotes that no significant alignment was found. % Id, percent identity; % cov, percent coverage. (207 KB DOC) [file pgen.0020182.st002.doc]

| Mouse |  |  |  |  |  |
| --- | --- | --- | --- | --- | --- |
| Query | Target Name | Target Start | Target End | % ID | % Coverage |
| UTX | chrX | 16401976 | 16516487 | 94 | 99 |
| DMD | chrX | 77797286 | 79851361 | 88 | 99 |
| NR0B1 | chrX | 80852459 | 80856248 | 61 | 100 |
| ZFX | chrX | 88739457 | 88759512 | 95 | 95 |
| EIF2S3 | chrX | 88851520 | 88873292 | 99 | 100 |
| PDHA1 | chrX | 153723191 | 153732638 | 99 | 82 |
| STS | * | * | * | * | * |
| RGN | chrX | 18789319 | 18800423 | 88 | 92 |
| PHF16 | chrX | 18718454 | 18756834 | 82 | 92 |
| RP2 | chrX | 18615821 | 18636673 | 83 | 84 |
| SLC9A7 | chrX | 18344906 | 18530625 | 92 | 96 |
| TUBGCP5 | chr7 | 50065373 | 50100363 | 88 | 93 |
| CYFIP1 | chr7 | 50141337 | 50199637 | 99 | 98 |
| NIPA2 | chr7 | 50202444 | 50214097 | 91 | 96 |
| NIPA1 | chr7 | 50248872 | 50289037 | 99 | 91 |
| UBE3A | chr7 | 53550079 | 53583181 | 95 | 88 |
| ATP10A | chr7 | 52936756 | 53106874 | 80 | 96 |
| GABRB3 | chr7 | 51863875 | 52097347 | 97 | 92 |
| GABRA5 | chr7 | 51680998 | 51763460 | 96 | 85 |
| GABRG3 | chr7 | 50993893 | 51659162 | 91 | 91 |
| OCA2 | chr7 | 50554068 | 50698884 | 76 | 69 |
| HERC2 | chr7 | 50320354 | 50500607 | 95 | 100 |
| APBA2 | chr7 | 58579102 | 58637178 | 91 | 88 |
| TJP1 | chr7 | 59181614 | 59238918 | 88 | 96 |
| KLF13 | chr7 | 57665288 | 57712324 | 86 | 61 |
| CHRNA7 | chr7 | 56872964 | 56985986 | 94 | 90 |
| CNGA3 | chr1 | 37548682 | 37556677 | 90 | 63 |
| INPP4A | chr1 | 37652393 | 37702731 | 94 | 98 |
| UNC50 | chr1 | 37725771 | 37733290 | 95 | 92 |
| MGAT4A | chr1 | 37739281 | 37830613 | 91 | 88 |
| SNRPB | chr2 | 129686454 | 129691020 | 99 | 95 |
| MKRN3 | chr7 | 56192363 | 56193527 | 63 | 66 |
| MAGEL2 | chr7 | 56153454 | 56154855 | 65 | 91 |
| NDN | chr7 | 56122145 | 56123120 | 77 | 93 |
| SNURF | chr7 | 53771930 | 53776009 | 97 | 96 |
| SNRPN | chr7 | 53759845 | 53764042 | 100 | 95 |
|  |  |  |  |  |  |
| Dog |  |  |  |  |  |
| Query | Target Name | Target Start | Target End | % ID | % Coverage |
| UTX | chrX | 38693898 | 38905089 | 98 | 99 |
| DMD | chrX | 26820708 | 28110606 | 93 | 100 |
| NR0B1 | chrX | 25349389 | 25354078 | 75 | 100 |
| ZFX | chrX | 19716698 | 19743870 | 97 | 96 |
| EIF2S3 | chrX | 19615905 | 19633540 | 100 | 100 |
| PDHA1 | chrX | 15291876 | 15305557 | 99 | 88 |
| STS | chrX | 4349468 | 4419017 | 79 | 90 |
| RGN | chrX | 40646435 | 40660867 | 91 | 91 |
| PHF16 | chrX | 40568419 | 40627708 | 89 | 96 |
| RP2 | chrX | 40420035 | 40468966 | 95 | 94 |
| SLC9A7 | chrX | 40193973 | 40336251 | 97 | 92 |
| TUBGCP5 | chr3 | 35126572 | 35185446 | 94 | 97 |
| CYFIP1 | chr3 | 34940370 | 35013332 | 96 | 98 |
| NIPA2 | chr3 | 34914424 | 34937719 | 98 | 100 |
| NIPA1 | chr3 | 34846702 | 34894912 | 99 | 92 |
| UBE3A | chr3 | 38254978 | 38290086 | 98 | 89 |
| ATP10A | chr3 | 37688624 | 37831089 | 93 | 95 |
| GABRB3 | chr3 | 36723892 | 36945197 | 91 | 93 |
| GABRA5 | chr3 | 36509909 | 36589323 | 96 | 92 |
| GABRG3 | chr3 | 35809726 | 36481382 | 98 | 89 |
| OCA2 | chr3 | 35208770 | 35558737 | 75 | 86 |
| HERC2 | chr3 | 34567357 | 34808560 | 97 | 100 |
| APBA2 | chr3 | 41224598 | 41285096 | 90 | 89 |
| TJP1 | chr3 | 41853070 | 41935256 | 93 | 97 |
| KLF13 | chr3 | 40494286 | 40494574 | 98 | 21 |
| CHRNA7 | chr3 | 39708369 | 39833183 | 97 | 91 |
| CNGA3 | chr10 | 47357444 | 47373494 | 80 | 78 |
| INPP4A | chr10 | 47212502 | 47267853 | 97 | 98 |
| UNC50 | chr10 | 47181239 | 47195374 | 98 | 92 |
| MGAT4A | chr10 | 47058833 | 47174295 | 95 | 89 |
| SNRPB | chr24 | 21673557 | 21679358 | 100 | 81 |
| MKRN3 | chr3 | 39273496 | 39274750 | 71 | 79 |
| MAGEL2 | chr3 | 39221582 | 39223139 | 82 | 98 |
| NDN | chr3 | 39172076 | 39172934 | 92 | 84 |
| SNURF | chr3 | 38706634 | 38711308 | 100 | 94 |
|  |  |  |  |  |  |
| Opossum |  |  |  |  |  |
| Query | Target Name | Target Start | Target End | % ID | % Coverage |
| UTX | scaffold_13259 | 897353 | 1147144 | 91 | 99 |
| DMD | scaffold_16798 | 2039723 | 4082158 | 80 | 99 |
| NR0B1 | scaffold_13277 | 3162209 | 3165883 | 68 | 49 |
| ZFX | scaffold_15001 | 6829094 | 6867590 | 94 | 95 |
| EIF2S3 | scaffold_15001 | 6661616 | 6676881 | 98 | 100 |
| PDHA1 | scaffold_14793 | 140165 | 152635 | 92 | 78 |
| STS | scaffold_13648 | 1529424 | 1631162 | 71 | 88 |
| RGN | scaffold_12679 | 1850467 | 1868249 | 79 | 87 |
| PHF16 | scaffold_12679 | 1893766 | 1943200 | 72 | 92 |
| RP2 | scaffold_12679 | 2134635 | 2152115 | 77 | 77 |
| SLC9A7 | scaffold_12679 | 2284724 | 2520910 | 89 | 90 |
| TUBGCP5 | scaffold_12679 | 2700968 | 2743015 | 83 | 90 |
| CYFIP1 | scaffold_12679 | 2817550 | 2912628 | 97 | 98 |
| NIPA2 | scaffold_12679 | 2917365 | 2923074 | 94 | 99 |
| NIPA1 | scaffold_12679 | 2960227 | 3019697 | 92 | 90 |
| UBE3A | scaffold_16658 | 2000681 | 2067334 | 94 | 88 |
| ATP10A | scaffold_16658 | 2589124 | 2884751 | 72 | 85 |
| GABRB3 | scaffold_16658 | 3943166 | 4204840 | 96 | 92 |
| GABRA5 | scaffold_16658 | 4432738 | 4551693 | 93 | 91 |
| GABRG3 | scaffold_12679 | 4693309 | 5071351 | 88 | 73 |
| OCA2 | scaffold_12679 | 3590688 | 4102983 | 88 | 61 |
| HERC2 | scaffold_12679 | 3169194 | 3373486 | 91 | 100 |
| APBA2 | scaffold_13378 | 2770235 | 2906606 | 77 | 85 |
| TJP1 | scaffold_13378 | 1854101 | 1931757 | 77 | 96 |
| KLF13 | scaffold_13378 | 3840428 | 3939057 | 57 | 61 |
| CHRNA7 | scaffold_13602 | 1306903 | 1511405 | 92 | 90 |
| CNGA3 | scaffold_16658 | 1923277 | 1939958 | 80 | 75 |
| INPP4A | scaffold_16658 | 953281 | 1042914 | 88 | 98 |
| UNC50 | scaffold_16658 | 747305 | 761216 | 95 | 92 |
| MGAT4A | scaffold_16658 | 619930 | 736494 | 80 | 88 |
| SNRPB | scaffold_15105 | 181332 | 190945 | 98 | 100 |
| MKRN3 | * | * | * | * | * |
| MAGEL2 | * | * | * | * | * |
| NDN | * | * | * | * | * |
| SNURF | * | * | * | * | * |
| SNRPN | scaffold_15105 | 160379 | 172878 | 94 | 100 |
|  |  |  |  |  |  |
| Tammar |  |  |  |  |  |
| Query | Target Name | Target Start | Target End | % ID | % Coverage |
| UBE3A | DQ985736 | 52 | 516 | 78 | 16 (Exons 9, 11) |
| GABRB3 | DQ985733 | 1 | 50 | 92 | 10 (Exon 4) |
| HERC2 | DQ985734 | 41 | 196 | 80 | 1 (Exon 20) |
| CNGA3 | DQ985737 | 1 | 213 | 95 | 7 (Exon 7) |
| SNRPB | AF176323 | 1 | 720 | 98 | 100 |
| SNRPN | DQ985732 | 1 | 720 | 95 | 100 |
|  |  |  |  |  |  |
| Platypus |  |  |  |  |  |
| Query | Target Name | Target Start | Target End | % ID | % Coverage |
| UBE3A | Oa_Bb_358D20 (AC153157) | 24936 | 62953 | 89 | 100 |
| CNGA3 | Oa_Bb_358D20 (AC153157) | 60943 | 55769 | 74 | 65 (Exons 5,6 and 7) |
| SNRPB | Contig_31468,  (AAPN01400224)  Contig_247728  (AAPN01346103) | 1558 (31468) | 413 (247728) | 95 | 56 (Exons 2, 5 and 6) |
|  |  |  |  |  |  |
| Chicken |  |  |  |  |  |
| Query | Target Name | Target Start | Target End | % ID | % Coverage |
| UTX | chr1 | 104551380 | 104664179 | 92 | 94 |
| DMD | chr1 | 108251626 | 109037065 | 70 | 98 |
| NR0B1 | chr1 | 109365081 | 109366342 | 70 | 25 |
| ZFX | chr1 | 111548006 | 111561852 | 88 | 95 |
| EIF2S3 | chr1 | 111583582 | 111596226 | 99 | 100 |
| PDHA1 | chr1 | 113631696 | 113639085 | 83 | 77 |
| STS | chr1 | 119318135 | 119402371 | 54 | 80 |
| RGN | chr1 | 123149368 | 123158318 | 82 | 71 |
| PHF16 | chr1 | 123169869 | 123187104 | 64 | 90 |
| RP2 | chr1 | 123236647 | 123241329 | 83 | 77 |
| SLC9A7 | chr1 | 123264670 | 123331702 | 83 | 84 |
| TUBGCP5 | chr1 | 123380151 | 123399712 | 81 | 93 |
| CYFIP1 | chr1 | 123419018 | 123469801 | 97 | 98 |
| NIPA2 | chr1 | 123472602 | 123476437 | 90 | 90 |
| NIPA1 | chr1 | 123491113 | 123494377 | 95 | 72 |
| UBE3A | chr1 | 124935062 | 124955276 | 93 | 88 |
| ATP10A | chr1 | 124712054 | 124820785 | 64 | 82 |
| GABRB3 | chr1 | 124459446 | 124487052 | 87 | 77 |
| GABRA5 | chr1 | 124292718 | 124339118 | 86 | 85 |
| GABRG3 | chr1 | 123988120 | 124283670 | 89 | 87 |
| OCA2 | chr1 | 123731609 | 123832426 | 75 | 55 |
| HERC2 | chr1 | 123531854 | 123635894 | 91 | 100 |
| APBA2 | chr10 | 6632517 | 6671352 | 72 | 85 |
| TJP1 | chr10 | 6801626 | 6842786 | 64 | 96 |
| KLF13 | chr10 | 6347546 | 6372312 | 51 | 61 |
| CHRNA7 | chr10 | 7435669 | 7473396 | 92 | 90 |
| CNGA3 | chr1 | 124962289 | 124963660 | 87 | 57 |
| INPP4A | chr1 | 125219580 | 125262427 | 88 | 98 |
| UNC50 | chr1 | 125338067 | 125341228 | 85 | 92 |
| MGAT4A | chr1 | 125347063 | 125416738 | 86 | 88 |
| SNRPB | chr20 | 10267880 | 10270086 | 96 | 95 |
| MKRN3 | * | * | * | * | * |
| MAGEL2 | * | * | * | * | * |
| NDN | * | * | * | * | * |
| SNURF | * | * | * | * | * |
| SNRPN | * | * | * | * | * |
|  |  |  |  |  |  |
| Zebrafish |  |  |  |  |  |
| Query | Target Name | Target Start | Target End | % ID | % Coverage |
| UTX | chr9 | 32971650 | 33010431 | 56 | 84 |
| DMD | chr1 | 4429041 | 4668091 | 13 | 95 |
| NR0B1 | * | * | * | * | * |
| ZFX | chr14 | 12381148 | 12382315 | 75 | 47 |
| EIF2S3 | chr24 | 11824002 | 11841685 | 98 | 95 |
| PDHA1 | chr5 | 12157945 | 12165204 | 79 | 78 |
| STS | * | * | * | * | * |
| RGN | chr6 | 26177135 | 26180169 | 70 | 47 |
| PHF16 | chrNA | 64322405 | 64339622 | 66 | 60 |
| RP2 | chr6 | 18690916 | 18691585 | 76 | 59 |
| SLC9A7 | chr6 | 18699088 | 18756493 | 78 | 78 |
| TUBGCP5 | chr6 | 18798400 | 18818005 | 64 | 80 |
| CYFIP1 | chr14 | 46365976 | 46403251 | 84 | 98 |
| NIPA2 | chr7 | 25441399 | 25450625 | 86 | 88 |
| NIPA1 | chr7 | 25453218 | 25456695 | 46 | 77 |
| UBE3A | chr6 | 21085748 | 21096430 | 83 | 88 |
| ATP10A | chr6 | 21017207 | 21028259 | 59 | 35 |
| GABRB3 | chr6 | 20568300 | 20659657 | 73 | 87 |
| GABRA5 | chr12 | 20349581 | 20376394 | 73 | 81 |
| GABRG3 | chr6 | 20780259 | 20937025 | 77 | 78 |
| OCA2 | chr6 | 21238248 | 21335124 | 70 | 52 |
| HERC2 | chr7 | 25475816 | 25552077 | 74 | 96 |
| APBA2 | chr7 | 23533804 | 23581194 | 67 | 80 |
| TJP1 | * | * | * | * | * |
| KLF13 | chr9 | 26874589 | 26896812 | 83 | 27 |
| CHRNA7 | chr7 | 58379186 | 58397973 | 67 | 81 |
| CNGA3 | chr6 | 21077771 | 21078989 | 83 | 50 |
| INPP4A | chr6 | 9301605 | 9358688 | 73 | 75 |
| UNC50 | * | * | * | * | * |
| MGAT4A | chr9 | 3708932 | 3759271 | 70 | 67 |
| SNRPB | chrNA | 8113050 | 8121800 | 93 | 57 |
| MKRN3 | * | * | * | * | * |
| MAGEL2 | * | * | * | * | * |
| NDN | * | * | * | * | * |
| SNURF | * | * | * | * | * |
| SNRPN | * | * | * | * | * |
