# Supplementary material for: Recent Assembly of an Imprinted Domain from Non-Imprinted Components
Source: PLoS Genet. 2006 Oct 27;2(10):e182. doi: 10.1371/journal.pgen.0020182 (PMC1626109; doi:10.1371/journal.pgen.0020182)
Supplement: Table S3 — (21 KB DOC) [file pgen.0020182.st003.doc]

| Species:Protein | Accession number |
| --- | --- |
| Mosquito:SmB | BX052864 |
| C.elegans:SmB | NM_060947 |
| Chicken:SmB | NM_204599.1 |
| Cow:SmB | NP_001029903 |
| Dog:SmN | XP_536165.1 |
| Drosophila:SmB | NM_057573.3 |
| Hedgehog:SmB | AF134826.1 |
| Honeybee:SmB | XM_392871 |
| Human:SmB | P14678 |
| Human:SmN | NP_003088 |
| Macaque:SmN | AB125194.1 |
| Mouse:SmB | NP_033251.1 |
| Mouse:SmN | NP_038698 |
| Opossum:SmB’ | AF134827 |
| Opossum:SmN | DQ899953 |
| Orangutan:SmN | CAH93359 |
| Platypus:SmB | AAPN01400224, AAPN01346103 |
| Rat:SmN | AAH87671 |
| Tammar:SmB | AF176323 |
| Tammar:SmN | DQ985732 |
| Tetraodon:SmB | CR721452.2 |
| Xenopus:SmB | BC041275.1 |
| Zebrafish:SmB | NM_205667.1 |
