# Supplementary material for: Recent Assembly of an Imprinted Domain from Non-Imprinted Components
Source: PLoS Genet. 2006 Oct 27;2(10):e182. doi: 10.1371/journal.pgen.0020182 (PMC1626109; doi:10.1371/journal.pgen.0020182)
Supplement: Text S1 — A primer extension assay was used to confirm the biallelic expression of UBE3A in platypus brain (Figure S2), because the polymorphism identified was a deletion and therefore difficult to test using direct sequencing. DNA was amplified in triplicate from genomic and cDNA samples extracted from the brain of three platypus individuals. One individual was homozygous for the C allele (I), another homozygous for the deleted allele (II), and a third heterozygous for both alleles (III). Each sample was subjected to a primer extension assay capable of quantifying the relative amount of each allele. In accordance with sequencing data, the heterozygous individual showed biallelic expression of the polymorphic site (Figure 1). Interestingly, there appeared to be an unexpected increase in the concentration of the deleted allele for all samples. This is most likely to be an experimental artifact, due to the polymorphism being located within a poly-C tract and resulting in primer slippage. (20 KB DOC) [file pgen.0020182.sd001.doc]

# SUPPLEMENTARY TEXT

# Expression studies

A primer extension assay was used to confirm the biallelic expression of *UBE3A* in platypus brain (Figure S2), because the polymorphism identified was a deletion and therefore difficult to test using direct sequencing. DNA was amplified in triplicate from genomic and cDNA samples extracted from the brain of three platypus individuals. One individual was homozygous for the C allele (I), another homozygous for the deleted allele (II), and a third heterozygous for both alleles (III). Each sample was subjected to a primer extension assay capable of quantifying the relative amount of each allele. In accordance with sequencing data, the heterozygous individual showed biallelic expression of the polymorphic site (Main text, Figure 1). Interestingly, there appeared to be an unexpected increase in the concentration of the deleted allele for all samples. This is most likely an experimental artefact, due to the polymorphism being located within a poly-C tract and resulting in primer slippage.
